# Practical Notes and Formulæ

**Published:** 1883-05-20

**Authors:** 


### PRACTICAL NOTES AND FORMULÆ.

**Cardiac Neurasthenia.**—In some cases of exhaustion from continuous overwork, the symptoms centre chiefly about the heart. The symptoms are feeble cardiac action, giddiness, weakness, intermittent heat. Palpitations, dyspnœa, and even syncope, may be present. A physician who suffered in this way for some time, writes to the British Medical Journal that he was relieved entirely by the following prescription :

| | | |
|---|---|---|
| ℞ | Quinin. sulph | grs. xxiv, |
| | Mist. camph, ad | ℥ vj, |
| | Acid hydrobromic, dil., | ʒ iij, |
| | Tinct. digital | ℥ ss, |
| | Liq. aurant | ℥ j, |
| | Tinct. nuc. vom | ʒ ij. |

M. Sig., ℥ ss three times a day —*Med. Record, Jan. 20.*

**Medication in Uterine Affections.**—Dr. J. Warren, Boston, recommends the following internal medication for relieving the engorged state of acute metritis :

| | | |
|---|---|---|
| ℞ | Chloral hydrat | ʒ iii, |
| | Chloral croton | gr. xxx, |
| | Liq. opii. comp | ʒ vi, |
| | Glycerinæ | ℥ ii, |
| | Syr. tolu | ℥ i. |

M. Teaspoonful every hour until ease from pain and sleep is produced.—*Kansas Med. Index.*

**Dysentery.**—The following is recommended as an excellent prescription for dysentery, particularly in children :

| | | |
|---|---|---|
| ℞ | Mono-bromated camphor | grs. xii, |
| | Sugar of milk | ʒ ij, |
| | Muriate morphine | grs. ii, |
| | Pulv. ipecac | grs. vi. |

M. Triturate thoroughly and divide into 24 powders. Give one to an adult every three hours, the bowels having first been evacuated with a dose of epsom salts.

To children reduce the dose according to age. If there be fever use the following—

| | | |
|---|---|---|
| ℞ | Tinc. aconite | 10 drops, |
| | Tinc. gelseminum | 30 drops, |
| | Water | 6 ounces. |

Give a teaspoonful every hour until the fever abates. W.

**Dysentery.**—If used early in the disease, the following will often cut short an attack of acute dysentery—

℞ Epsom salts........................... 2 drachms,
Water................................ 6 ounces,
Tinct. aconite.......................... 10 drops,
Muriate morphine..................... 1 grain.

M. A tablespoonful every two hours until the character of the discharges change, then continue in reduced doses from 24 to 48 hours, after which the bowels may be checked with burnt toddy and paregoric or other opiate. Sometimes the distress is greatly aggravated and prolonged by a high degree of inflammatory sensibility in the rectum, in which case wash out the bowel with a free injection of tepid water, which follow with an anodyne enema of starch-water 2 ounces, muriate morphine ⅓ grain. W.

**Remedy for Constipation.**—

℞ Pulv. aloes.............................. 30 grains,
Ext. belladonnæ, fl......................... 20 minims,
Ext. nucis vom fl.......................... 30 minims,
Pulv. ipecac............................... 3 grains,
Tinct. gentian comp....................... 2 ounces,
Syr. simp. to make........................ 4 ounces.

M. Sig. Teaspoonful on the evening of each day when the bowels have not moved. The dose is for adults. For children, five drops for each year of age.—*Drug. Circular.*

**Bilious Dysentery.**—This is a form of the disease which frequently occurs in hot, malarious districts, characterized by a bilious and disordered state of the stomach and secretions, sometimes nausea and vomiting, and a remittent form of fever, with the usual dysenteric discharges. Here the treatment most available is the following—

℞ Calomel.................................... grs. vi,
Dover's powder............................ grs. x.

M. Make three powders. Give one every three hours until all are taken, and two hours after last dose give one drachm of epsom salts. After it has operated follow with quinine and Dover's powder in sufficient doses, at intervals of three hours, from 6 o'clock a. m. till 3 o'clock p. m., and give weak solution of aconite and gelseminum if there be fever, as follows—

℞ Tinc. aconite.............................. 10 drops,
Water..................................... 6 ounces,
Gelseminum.............................. 20 drops.

M. Give a teaspoonful every hour until the fever abates. Usually the fever drops are required only during the evening. W.

**Novel Method of Taking Quinine.**—Dr. J. T. Stoddard writes: Take a portion of the white of an egg, say half teaspoonful, put it in a teaspoon. Take your dose of quinine—whatever amount you intend to give—press it firmly in a fold of paper between your thumb and finger. Now, telling an assistant to hold the spoon, let the quinine slide out of the paper into the spoon, taking care not to disturb the quinine. Take enough of the albumen to cover the quinine. Now dip the spoon, holding it level, into hot water only for a moment, to color the egg, then into cold water, and offer your patient soft boiled egg, and they will take it nine times out of ten without knowing that there is quinine in it.

A medical friend of Texas, whose name has escaped us, sends the following—

℞ Quiniæ sulph ʒ i,

Potass. bromid ℥ i,

Tinct. ferri mur. ʒ iij,

Aq. puræ ad ℥ xvi.

M. ft. sol. S. Tablespoonful every two hours until 6 doses are taken as a chill preventive. A tablespoonful after each meal as a tonic.

**Acute Bronchitis.**—The late Prof. Joseph Pancoast used in private practice with much success the following mixture for acute cases of bronchitis:

℞ Pruni virg. cort } aa ʒ iv

Senegæ rad }

Ipecac, rad ʒ ij,

Ext. conii gr. xv,

Aquæ q. s. ft. (by displacement) ℥ viij.

Then add—

Spts. genevæ ℥ j,

Tinct. cardamon, comp ℥ j.

M. S. Two teaspoonfuls in water whenever troubled with cough.—*Medical Bulletin.*

**Diarrhœa.**—In a case of mucous diarrhœa in a child one year of age, Dr. Bruen prescribed what he called his favorite prescription:

℞ Bismuth. subnit gr. lx,
Fl. ext. rhubarb gtt. viij,
Syrup blackberry fl. ℥ ss,
Elixir orange fl. ℥ ss.

M. Of this the child was ordered to take a teaspoonful four to six times a day. Proper feeding—barley-water, milk, and lime-water, was also directed. Starchy food was positively prohibited. —*Louisville Med. News.*

